# Are Yellow Sticky Traps an Effective Method for Control of Sweetpotato Whitefly, *Bemisia tabaci*, in the Greenhouse or Field?

**DOI:** 10.1673/031.012.11301

**Published:** 2012-10-06

**Authors:** Yaobin Lu, Yawei Bei, Jinming Zhang

**Affiliations:** State Key Laboratory Breeding Base for Zhejiang Sustainable Pest and Disease Control, Institute of Plant Protection and Microbiology, Zhejiang Academy of Agricultural Sciences, Hangzhou Zhejiang 310021, China

**Keywords:** entire crop growth period, population dynamics

## Abstract

Yellow sticky traps are a common method for monitoring many pests, but it has not been shown whether they could be used as a control method. In this study the impact of yellow sticky traps on the population dynamics of the sweetpotato whitefly, *Bemisia tabaci* (Gennadius) (Hemiptera: Aleyrodidae) was determined in the greenhouse and field. In the greenhouse, yellow sticky traps significantly suppressed the population increase of adult and immature whiteflies. The whitefly densities in the greenhouse with traps were significantly lower than the greenhouse without traps. In the field, traps did not have a significant impact on the population dynamics of adult and immature whiteflies. The densities in fields with traps were very similar to fields without traps. These results suggest that yellow sticky traps can be used as an effective method for the control of whiteflies in the greenhouse, but not in the field. This information will prove useful for the effective management of whiteflies in greenhouses.

## Introduction

Sweetpotato whitefly *Bemisia tabaci* (Gennadius) (Hemiptera: Aleyrodidae) was first recorded in Greece in 1889 (Gennadius 1889). It has become one of the most serious agricultural pests in many areas of the world in recent decades. This pest can cause damage through feeding, causing sooty mold by its honeydew, transmitting more than 111 species of plant-pathogenic viruses, and inducing plant physiological discords (Brown 1994; Heinz 1996; Jones 2003). The worldwide losses caused by this pest exceed $300 million annually (De Barro and Driver 1997; White 1998). The actual number of biotypes of this pest is unknown, but at least 24 different biotypes have been identified (Perring 2001), of which biotype B is the most serious and widely distributed one. *Bemisia tabaci* biotype B, which is synonymous with *Bemisia argentifolii* Bellows and Perring, has a wide range of hosts, attacking more than 500 different species of plants including fruits, vegetables, fiber, and ornamental crops (Greathead 1986). In past 20 years, biotype B has spread rapidly around the world to become a major crop pest in tropical and subtropical regions (Brown et al. 1995; Oliveira et al. 2001; Rekha et al. 2005).

In China, *B. tabaci* was first recorded in 1949 (Luo et al. 1989), has been a serious pest since the mid-1990s, and has been found in more than 20 provinces in China (Luo and Zhang 2000; Ren et al. 2001). There are about 74 reported species of host plants of *B. tabaci* in Beijing (Luo et al. 2000) and 176 species in Guangzhou (Qiu et al. 2001). Biotype B is also the most serious biotype in most regions of China and has caused tremendous losses (Luo et al. 2002; Lin et al. 2003; Qiu et al. 2003; Wu et al. 2003; Li et al. 2005; Zang et al. 2005; Chu et al. 2006).

*Bemisia tabaci*, especial biotype B, is difficult to control because of its high resistance to many insecticides available in the market, its wide range of hosts, and rapid rate of development and reproduction (De Barro and Driver 1997; He and Huang 2005). In China, the control of *B. tabaci* mainly relies on chemical insecticides, which has caused many serious problems (Ren et al. 2001). It is necessary to explore some non-chemical methods to control this pest effectively and to significantly reduce the spray of chemical insecticides.

Yellow sticky traps are a commonly used method for population monitoring of many pests. In recent decades, studies of these traps mainly focused on how to use them to monitor populations of pest species such as whiteflies, leafminers, and aphids (Berlinger 1980; Byrne et al. 1986; Shen and Ren 2003; Zhou et al. 2003; Qiu et al. 2006; Gu et al. 2008). In recent years, yellow sticky traps have also been used as a method for the control of some pests, especially for the control of whitefly. The combination of yellow sticky traps and parasitoids has proven to be an effective method for the control of *B. tabaci* in a greenhouse (Shen and Ren 2003; Gu et al. 2008). Abdel-Megeed et al. (1998) also demonstrated that for control purposes, yellow sticky traps can significantly reduce the density of *B. tabaci* in field. But all these mentioned studies about the effect of traps on whitefly were conducted during only part of a crop's growing period. Thus, it is unknown if yellow sticky traps are an effective method for whitefly control for the entire crop growth period from transplantation to harvest. Additionally, all aforementioned studies were done in a single season, and thus it is unknown if these traps are effective over several seasons. Therefore, the objective of this study was to determine the impact of yellow sticky traps on the population dynamics of *B. tabaci* through the whole growth period of crops and over several years, in order to determine if these traps are an effective control for this key pest in the greenhouse and in the field.

## Materials and Methods

### Yellow sticky trap, host plant, and *B. tabaci*

The yellow sticky trap was made of art paper (10 × 30 cm, 250 g/m^2^). The art paper was painted lemon yellow on both sides, sealed with a thin transparent plastic cover, and smeared with sticky glue. During the experiments, the traps were hung about 30 cm above the crop canopies and could be adjusted vertically whenever the crop attained additional growth.

Eggplant, *Solanum melongena* L. (Solanales: Solanaceae) was used as the host plant of *B. tabaci*, because eggplant is one of the most favorable host plants for *B. tabaci* and is also an important economic crop in China. It was therefore selected as a representative host plant in this report. Eggplants were seeded in plastic trays and allowed to grow up to the 4–6 leaf stage in a pest-free greenhouse. Plants were then transplanted into either greenhouse or field for trials.

Some eggplant leaves with eggs and larvae of *B. tabaci* were collected from the eggplant field in the suburb of Hangzhou, China and reared on eggplants in a temperaturecontrolled room. When the pupae emerged, the adults were collected for trials.

### Trapping trials

Trapping studies were conducted concurrently for eggplants growing in both the greenhouse and the field. Four treatments were designed for this experiment: (1) greenhouse with yellow sticky traps, (2) greenhouse without traps, (3) field with yellow sticky traps, and (4) field without traps. Each treatment had three replications. Six greenhouses (6 × 30 m) and six field plots (6 × 30 m) 100 m apart from each other were selected for trials in the suburb of Hangzhou, China. Crops near the selected greenhouses and fields were growing the same species of eggplant. First, eggplants with 4–6 leaves were transplanted into the selected greenhouses and fields. Then, yellow sticky traps were uniformly hung in three selected greenhouses and three fields at the rate of 1 trap/5 m^2^ (about 36 traps in one greenhouse or one field). The traps were removed from greenhouses and fields every seven days and replaced by new ones. There were no traps in control greenhouses and fields. In order to ensure the original whiteflies in each greenhouse and field were similar, about 5000 *B. tabaci* adults (♂:♀ = 1:1) were uniformly released in each greenhouse and field at the time when traps were hung. Each greenhouse or field only had one release of *B. tabaci* adults during the experimental period. Fifteen days after the release of whitefly adults into selected greenhouses and fields, whitefly adults and immatures (eggs, larvae, pupae) on leaves were counted once every week until the fruits were harvested completely. For each counting period, 50 leaves in each greenhouse and field were randomly selected but not cut away from plant, and the adults on each leaf were carefully and quickly counted. In addition, 50 leaf discs (1 cm^2^) in each greenhouse and field were taken into the laboratory, and immatures on each disc were checked under a Nikon SMZ1500 stereomicroscope (www.nikon.com). This study was conducted three separate times: 7 June to 6 September 2005; 12 April to 25 July 2006; and 15 August to 10 October 2007 respectively. All studies were conducted at the same place. During the trials no insecticides were applied in greenhouses or fields.

### Experimental design and data analysis

Completely randomized design was used to arrange experimental replications of different treatments in greenhouses and fields. Data from each treatment on each checking day were averaged. Then a *t*-test for two independent samples was used to test the significance between the means of different treatments on the same date. All calculations were performed using Statistica (StatSoft Inc., www.statsoft.com).

## Results

### Population dynamics of adult whiteflies

**Figure 1.**
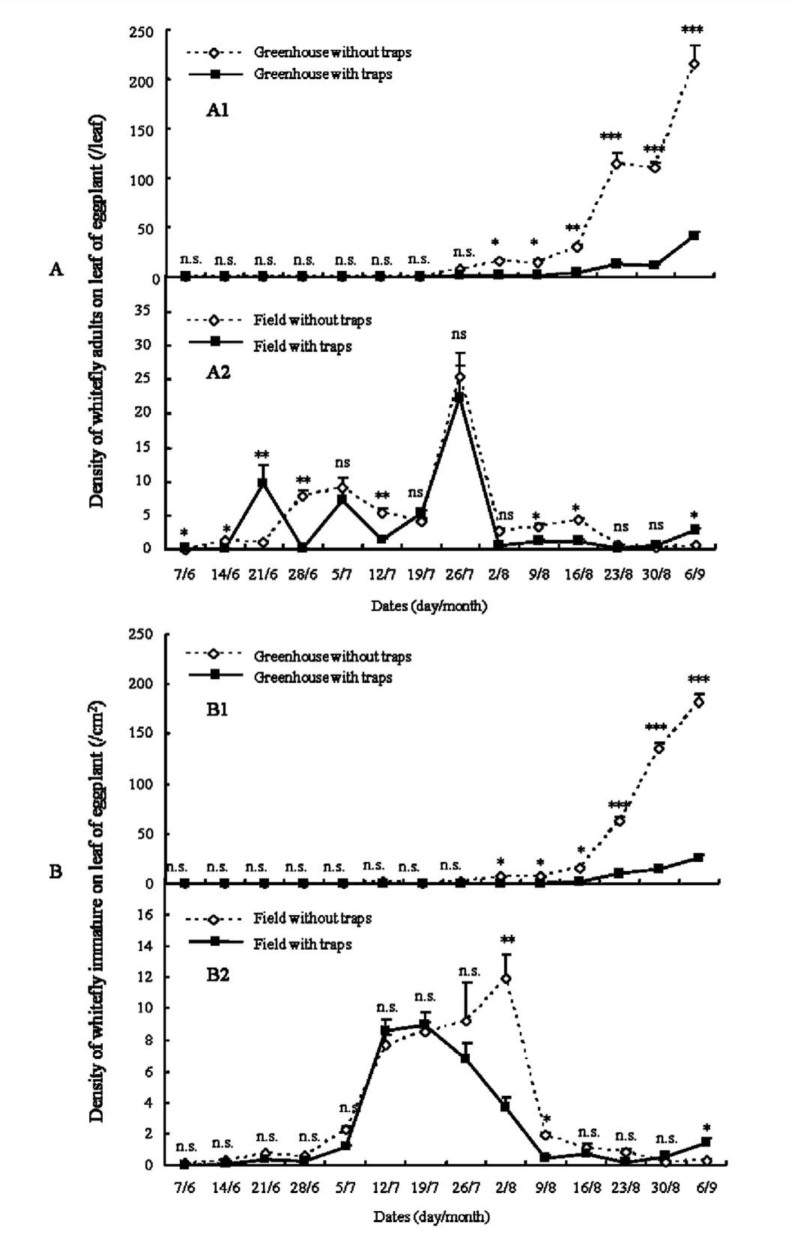
The population dynamics of *Bemisia tabaci* adult (A) and immature (B) on eggplant from 7 June to 6 September in 2005 (Asterisks (^*^) in A1, A2, B1, and B2 indicate statistically significant difference between the two treatments respectively, n = 3: ^***^p < 0.001, ^**^p < 0.01, ^*^p < 0.05, n.s. = not significant). High quality figures are available online.

**Figure 2.**
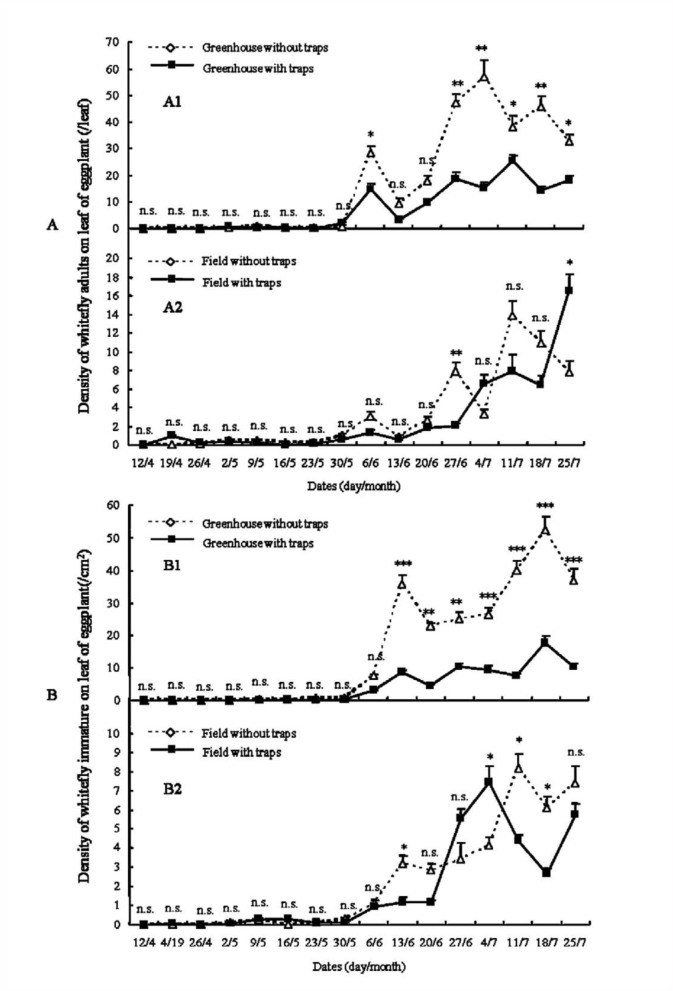
The population dynamics of *Bemisia tabaci* adult (A) and immature (B) on eggplant from 13 April to 25 July in 2006 (Asterisks (^*^) in A1, A2, B1, and B2 indicate statistically significant difference between the two treatments respectively, n=3: ^*^p < 0.001,^ *^p < 0.01, ^*^p < 0.05, n.s. = not significant). High quality figures are available online.

**Figure 3.**
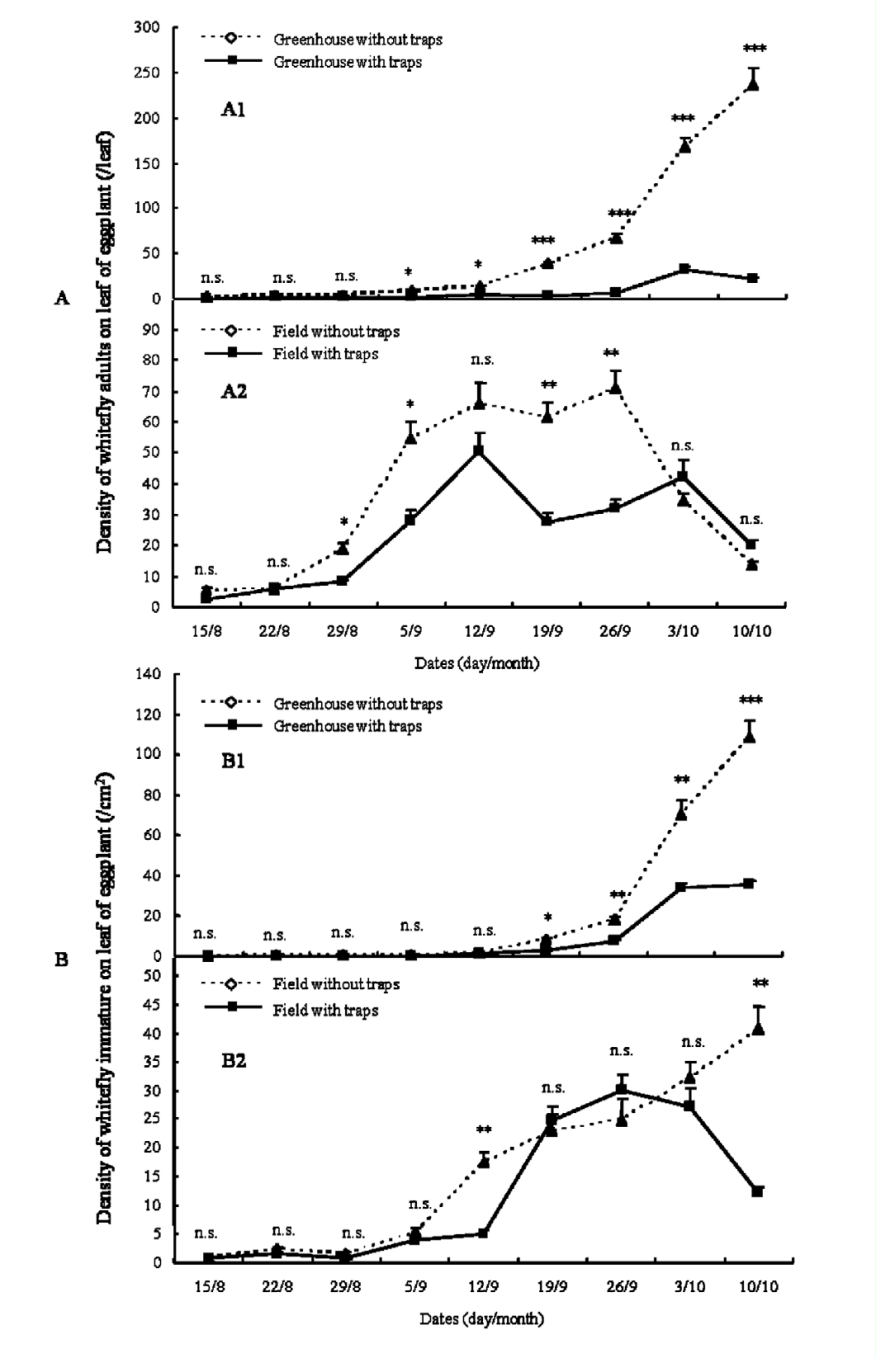
The population dynamics of *Bemisia tabaci* adult (A) and immature (B) on eggplant from 15 August to 17 October in 2007 (Asterisks (^*^) in A1, A2, B1, and B2 indicate statistically significant difference between the two treatments respectively, n=3: ^*^p < 0.001, ^**^p < 0.01, ^*^p < 0.05, n.s. = not significant). High quality figures are available online.

Yellow sticky traps had very different impacts on the dynamics of adult whiteflies in greenhouse and field (Figure 1A, Figure 2A, Figure 3A).

In the greenhouse, yellow sticky traps significantly suppressed the increase of adult density (Figures 1A1, 2A1, 3A1). In the first trial, from 7 June to 6 September 2005 (Figure 1A1), whitefly adult densities in greenhouses with traps in the early period of trial (7 June to 26 July) were not significantly different from control greenhouses without traps. But the adult densities of controls at different dates of the late period of the trial (2 August to 6 September) were significant higher than greenhouses with traps (*p*_2/8_ < 0.05, *p*_9/8_ < 0.05, *p*_16/8_ < 0.01, *p*_23/8_ < 0.01, *p*_30/8_ < 0.01, *p*_6/9_ < 0.01. The subscript values indicate the trial date, i.e., day/month, on which the samples were collected). In the second trial, from 12 April to 25 July 2006 (Figure 2A1), the adult densities during 12 April to 30 May were very low and not significantly different between control greenhouses and greenhouses with traps. From 6 June to 25 July, the adult densities of controls were significantly higher than greenhouses with traps (*p*_6/6_ < 0.05, *p*_27/6_< 0.01, *p*_4/7_ < 0.01, *p*_11/7_ < 0.05, *p*_18/7_ < 0.01, *p*_25/7_ < 0.05), except on 14 June and 20 June (*p* > 0.05). In the third trial, from 15 August to 10 October in 2007 (Figure 3A1), the results were very similar to the trial in 2005. The adult densities at 15 August, 22 August, and 29 August were not significantly different between control greenhouse and those with traps. The adult densities in greenhouses with traps during 5 September to 10 October were significantly lower than in controls (*p*_5/9_ < 0.05, *p*_12/9_ < 0.05, *p*_19/9_ < 0.01, *p*_26/9_ < 0.01, *p*_3/10_ < 0.01, *p*_10/10_ < 0.01).

In the field, yellow sticky traps did not have obvious impact on adult dynamics (Figures 1A2, 2A2, 3A2). The dynamics of whitefly adults in control fields and fields with traps were very similar. In the trial from 7 June to 6 September 2005 (Figure 1A2), the adult densities in fields with traps at some dates (7 June, 21 June, 6 September) were significant higher than controls (*p*_7/6_ < 0.05, *p*_21/6_ < 0.01, *p*_6/9_ < 0.05), but at other dates (14 June, 28 June, 12 July, 9 August, 16 August) were significant lower than controls (*p*_14/6_ < 0.05, *p*_28/6_ < 0.01, *p*_12/7_ < 0.01, *p*_9/8_ < 0.05, *p*_16/8_ < 0.05). At all remaining dates (5 July, 19 July, 26 July, 2 August, 23 August, 30 August), adult densities in fields with traps were not significantly different than controls. In the trial from 13 April to 25 July 2006, adult densities at most dates were not significantly different between fields with traps and controls. In the trial from 15 August to 10 October 2007, adult dynamics of fields with traps was similar to controls.

### Population dynamics of immature whiteflies

The dynamics of immature whiteflies was similar to that of adults described previously (Figures 1B, 2B, 3B). Yellow sticky traps also had very different impacts on the population dynamics of the immature whiteflies under greenhouse and field conditions.

In the greenhouse, densities of immature whiteflies were significantly reduced by yellow sticky traps (Figures 1B1, 2B1, 3B1). In the trial from 7 June to 6 September 2005 (Figure 1B1), the immature densities in the early period (7 June to 26 July) were not significantly different between control greenhouses and those with traps, but densities in greenhouses with traps became significantly lower than controls in the late period (2 August to 6 September) (*p*_2/8_ < 0.05, *p*_9/8_ < 0.05, *p*_16/8_ < 0.05, *p*_23/8_ < 0.01, *p*_30/8_ < 0.01, *p*_6/9_ < 0.01). In the trials from 13 April to 25 July 2006 and from 15 August to 10 October 2007, similar results were obtained (Figures 2B1 and 3B1). The densities of immature whiteflies in greenhouses with traps during 13 June to 25 July 2006 and 19 September to 10 October 2007 were significant lower than controls (13 June to 25 July: *p*_13/6_ < 0.01, *p*_20/6_ < 0.01, *p*_27/6_ < 0.01, *p*_4/7_ < 0.01, *p*_11/7_ < 0.01, *p*_18/7_ < 0.01, *p*_25/7_ < 0.01; 19 September to 10 October: *p*_9/9_ < 0.05, *p*_26/9_ *<* 0.01, *p*_3/10_ < 0.01, *p*_10/10_ < 0.01).

In the field the impact of yellow sticky traps on immature whitefly dynamics was not obvious (Figures 1B2, 2B2, 3B2). In the trial from 7 June to 6 September 2005 (Figure 1B2) the dynamics of immature in controls and those with yellow sticky traps were very similar. Densities in fields with traps on most dates were not significantly different from controls; only densities on 2 August, 9 August, and 6 September were significantly different (*p*_2/8_ < 0.01, *p*_9/8_ < 0.05, *p*_6/9_ < 0.05). In the trials from 13 April to 25 July 2006 (Figure 2B2) and from 15 August to 10 October 2007 (Figure 3B2), the immature dynamics in fields with traps were not significantly different from controls.

## Discussion

Yellow sticky traps have been used as a control method for whiteflies in greenhouses and in the field for many years. But according to our knowledge, all prior studies were done in a short time period and not throughout the entire growth period of crop. For example, Gu et al. (2008) evaluated the impact of yellow sticky traps on the population suppression of *B. tabaci* in the greenhouse. Their experiments lasted 40 days from 18 July to 22 August 2005. Our experiments were done over three years covering the entire growth period from transplantation to harvest, demonstrating that yellow sticky traps are an effective method for whitefly control at the different stages of crop growth in the greenhouse. In addition, according to our knowledge, previous studies of the use of yellow sticky traps for whitefly control in the field were conducted with variation in the original number of pests, which would be an unreliable method for showing a significant difference between treatment and control resulting from the effect of traps (Abdel-Megeed et al. 1998; Kim et al. 1999; Gu et al. 2008).

Our study over three years showed that yellow sticky traps can significantly suppress the population increase of adult and immature whiteflies in the greenhouse. But in the field, traps could not significantly prevent the increase of a whitefly population.

There are two reasons that potentially explain why the yellow sticky traps in the greenhouse effectively suppressed whitefly populations. First, since yellow sticky traps can capture a great number of whitefly adults and reduce the adults on host plants, fewer eggs were laid on host plant leaves and fewer larvae could be found on leaves. Second, the greenhouse was covered with plastic film and nylon mesh, which could significantly reduce the migration of whitefly adults between different greenhouses, and between greenhouse and field. Thus, yellow sticky traps were the main factor causing the significant difference of population densities between greenhouses with traps and greenhouses without traps; the whitefly population density in greenhouses with yellow sticky traps was kept significantly lower than in greenhouses without traps.

In the field there were also two factors that potentially led to the failure of yellow sticky traps to control whitefly populations. One may be that although traps captured many whitefly adults and reduced the adults on host plants in the field, many adults in other neighboring fields could have migrated to the experimental field. The other potential factor may be that yellow sticky traps were not only attractive to whitefly adults in the experimental field but also to adults in other fields. Thus, many adults in other fields may have actually been attracted to migrate specifically to the experimental field. Therefore, some adults were captured by yellow sticky traps and others landed on the plants within experimental fields.

From this study, two reasonable suggestions for the utilization of yellow sticky traps in the greenhouse and the field can be made. First, yellow sticky traps can be used to effectively monitor and control *B. tabaci* in the greenhouse. Second, in the field, yellow sticky traps can be used as a monitoring method, but not for control.

Although yellow sticky traps significantly suppressed the population in terms of rate of increase in the greenhouse, in most cases the population density of *B. tabaci* was still relatively higher than the economic thresholds. According to Shen et al. (2005) the threshold for *B. tabaci* on eggplant is 4.6119 adults per leaf. But in our study, although yellow sticky traps significantly suppressed the population increase in the greenhouse, adult density on most dates significantly exceeded this threshold. Thus, in order to control the pest density under the economic threshold, yellow sticky traps should be used in conjunction with other suitable methods, such as biological control, cultural control, and selective insecticides. In China, chemical control is still the main method for the control of *B. tabaci* and other pests in the greenhouse (Lin et al. 2003). This study did not evaluate the effect of the combinations of yellow sticky traps and insecticides on the population dynamics of *B. tabaci*. Further studies are needed to find out how to best use yellow sticky traps and pesticides together, and to test how different combinations might significantly reduce the use of these pesticides.

Climatic factors such as temperature, wind, rain, and relative humidity, as well as natural enemies, play important roles in the population dynamics of whiteflies (Byrne 1991). Comparing the population dynamics of *B. tabaci* (Figures 1, 2, 3), the trends of the population dynamics of adult and immmature whiteflies in greenhouses in different years were very similar, but the trends in fields in different years varied greatly. These phenomena may be caused by climatic factors and natural enemies. In the greenhouse, values of climatic factors and the abundance of natural enemies in different years were similar, which led to the similar trends of the population dynamics. But in the field, climatic factors and natural enemies varied greatly in different years, which may be the main factors that caused the very different trends of population dynamics in different years. In addition, the population densities of adult and immature whiteflies in greenhouses without traps in different years were significant higher than fields without traps (Figures 1, 2, 3), which may also have been caused by climatic factors and natural enemies.
